# Lagged symptom-level associations among pain, sleep disturbance, and depressive-affective symptoms in adults aged ≥50 years: an international longitudinal multi-cohort study

**DOI:** 10.3389/fmed.2026.1921405

**Published:** 2026-08-21

**Authors:** Zeng Chen, Jianqiang Wang, Xiaoqing Chen, Sheng Hu

**Affiliations:** 1 College of Sports Medicine, Wuhan Sports University, Wuhan, China; 2 Department of Rehabilitation Medicine, Geriatric Hospital of Hainan, Haikou, Hainan, China

**Keywords:** aging, cross-lagged panel network, depressive symptoms, international multi-cohort study, lagged association, pain, sleep disturbance

## Abstract

**Background:**

Pain, sleep disturbance, and depressive-affective symptoms commonly co-occur in adults aged ≥ 50 years, but their temporal ordering at the individual symptom level remains unclear. We estimated lagged symptom-level associations across five international aging cohorts and quantified heterogeneity between cohorts.

**Methods:**

We analyzed harmonized longitudinal data from five aging cohorts with repeated pain and affective symptom measures: ELSA, HRS, KLoSA, MHAS, and SHARE. CHARLS and LASI were excluded due to the absence of repeated direct pain measures or insufficient longitudinal waves. Participants contributed complete adjacent-wave observations when six binary symptom nodes were measured at waves t and t + 1. Cohort-specific weighted logistic cross-lagged panel network models adjusted for lagged symptoms, sociodemographic covariates, wave indicators, participant clustering, survey weights, and inverse probability of attrition weights. Directed network edges were pooled using REML random-effects meta-analyses with Benjamini–Hochberg false discovery rate correction. A five-node sensitivity analysis excluded the low positive affect node.

**Results:**

The analysis included 123,608 participants contributing 405,472 complete adjacent-wave transitions. Sleep disturbance was the most precise pooled lagged predictor of later depressed mood (OR 1.41, 95% CI 1.36–1.46). Pain predicted later sleep disturbance, fatigue/effort, depressed mood, and loneliness, whereas sleep disturbance and fatigue/effort bidirectionally predicted later pain. Several clinically relevant associations were statistically significant but highly heterogeneous, most notably pain predicting sleep disturbance (I^2^ = 96.8%), pain predicting fatigue/effort (I^2^ = 96.9%), and loneliness predicting depressed mood (I^2^ = 92.5%). These associations remained statistically significant in Hartung–Knapp sensitivity analyses, though prediction intervals indicated substantial between-cohort variation. The five-node sensitivity analysis yielded similar estimates for all main pathways.

**Conclusion:**

Pain, sleep disturbance, loneliness, fatigue/effort, and depressive-affective symptoms showed consistent prospective lagged associations across five international aging cohorts, though effect sizes varied substantially across populations. These findings can inform joint symptom assessment and identify promising symptom pairs for future within-person and causal-inference research. However, they do not establish direct causal symptom dynamics, actionable intervention targets, or country-level differences.

## Introduction

Pain, sleep disturbance, and depressive symptoms are common and clinically significant in adults aged ≥50 years (1, 2). Their co-occurrence compounds symptom burden, complicates chronic disease management, and compromises functional independence, rehabilitation engagement, and quality of life (3). Mechanistically, sleep disturbance, loneliness, fatigue, and depressive symptoms share biological, behavioral, and social pathways in later life (4, 5). While longitudinal studies have demonstrated bidirectional or mutually predictive associations between pain, sleep disturbance, and depressive symptoms in community aging cohorts (6, 7), recent network and symptom-cluster studies further suggest pain, sleep problems, fatigue, loneliness, and depressed mood form temporally interconnected networks (8, 9).

The majority of longitudinal evidence has relied on total symptom scores, symptom counts, or single exposure-outcome models. Although these approaches estimate average associations, they cannot identify which specific symptom at one wave predicts another symptom at the next after accounting for symptom persistence and co-occurring symptoms. In contrast, symptom network theory conceptualizes mental and somatic symptoms as interacting components rather than interchangeable indicators of a single latent condition (10, 11). Cross-lagged panel network models extend this framework to longitudinal cohort data by estimating directed symptom-level predictive pathways (12). However, empirical evidence on symptom-level lagged associations evaluated across multiple international aging cohorts remains limited.

International multi-cohort studies can determine whether lagged symptom-level associations are consistent across aging cohorts. While harmonized aging data resources provide repeated measures for such comparisons, cohort-level differences may reflect both contextual and measurement variation rather than true country-level effects (13). This distinction is central to study interpretation: although pooled estimates can identify shared predictive patterns and quantify between-cohort variability, they cannot evaluate direct national differences.

We conducted an international longitudinal multi-cohort study to estimate lagged symptom-level associations between pain, sleep disturbance, and depressive-affective symptoms in adults aged ≥ 50 years. We specifically aimed to (1) identify directed lagged associations, (2) quantify cross-cohort heterogeneity, and (3) evaluate exploratory network summaries. We hypothesized that pain would predict later sleep disturbance and fatigue/effort, sleep disturbance would predict later depressed mood, and the network structures would reveal both shared associations and cohort-specific variation. In addition, this study was designed to integrate clinically relevant symptom pairs—including pain–sleep, sleep–mood, pain–fatigue, and loneliness–mood pathways—into a single longitudinal network framework to guide future within-person and causal-inference research.

## Methods

### Study design and participants

We conducted a secondary analysis of de-identified harmonized longitudinal aging cohort data in accordance with STROBE reporting guidelines for observational studies. The analysis estimated prospective lagged predictive associations between symptom nodes across adjacent survey waves. These estimates reflect conditional population-level associations adjusted for prior symptoms and covariates but do not establish within-person causal symptom dynamics.

#### Data sources

We audited seven downloaded databases: HRS, ELSA, SHARE, KLoSA, MHAS, CHARLS, and LASI. The final analysis used five international cohorts with repeated direct pain and symptom items: ELSA, HRS, KLoSA, MHAS, and SHARE. These cohorts are part of the HRS-family or related international aging-study infrastructure that supports harmonized aging research (14, 15). We excluded CHARLS from the longitudinal CLPN models because the downloaded Harmonized CHARLS file contained repeated CES-D-like symptom items but did not contain a repeated direct pain item. Similarly, we excluded LASI because the available Harmonized LASI file contained only one wave, precluding lagged network estimation. SHARE was analyzed as one cohort rather than as country-disaggregated data; therefore, heterogeneity analyses are interpreted as cross-cohort rather than country-level comparisons.

#### Study population

Eligible observations were from participants aged ≥ 50 years with at least one adjacent pair of waves. For the primary common-network analysis, pain, sleep disturbance, depressed mood, loneliness, fatigue/effort, and low positive affect were required to be measured at both wave t and wave t + 1. This complete six-node transition design ensured a common analytic matrix across all directed edges, though it was conservative because a transition could be excluded due to missing future symptoms that were not the outcome in a specific model. Because survey intervals differed across cohorts, estimated edges are interpreted as average associations across adjacent survey waves rather than effects over a fixed time interval. Differences in elapsed time may influence symptom transition probabilities and represent a potential contributor to between-cohort heterogeneity. Individuals could contribute multiple adjacent-wave transitions. The adjacent-wave transition served as the primary analytic unit, with standard errors clustered by participant.

### Assessment of symptom nodes

Six binary nodes were harmonized across cohorts: pain, sleep disturbance, depressed mood, loneliness, fatigue/effort, and low positive affect. Pain was defined using repeated direct pain-frequency or pain-problem items and does not necessarily signify chronic pain. Sleep disturbance was defined using restless sleep or EURO-D sleep items and does not denote clinical insomnia. Depressed mood was derived from felt-depressed or EURO-D depression items and does not represent major depressive disorder. Loneliness was operationalized via felt-lonely items or, in SHARE, a three-item loneliness mean score dichotomized at ≥2. Fatigue/effort was captured using everything-an-effort or EURO-D fatigue items. Positive affect items, including feeling happy or enjoying life, were reverse-coded to indicate low positive affect. Frequency-coded negative symptoms were classified as present at ≥2. For KLoSA, the CES-D happy item was coded 1 = very rarely, 2 = sometimes, 3 = often, and 4 = almost always. To improve comparability with binary happy/enjoyment items in HRS, ELSA, MHAS, and SHARE, low positive affect in KLoSA was defined solely as a response of “very rarely happy.” Because item wording, response options, and dichotomization thresholds differed across cohorts, measurement non-equivalence remains an important limitation. Although harmonization improved cross-cohort comparability, it inevitably introduced measurement uncertainty that may contribute to observed heterogeneity. Source variables and dichotomization rules are detailed in Supplementary Table S1A.

### Assessment of covariates

Models were adjusted for age, sex, education, chronic disease count, and survey wave. Education was harmonized within each cohort and entered as a cohort-specific covariate; consequently, it was not treated as a directly comparable cross-cohort exposure. Chronic disease count included self-reported hypertension, diabetes, heart disease, stroke, cancer, chronic lung disease, and arthritis where available. Cohort-specific person-level survey weights were scaled to a mean of 1. Although physical activity is considered a potentially important time-varying behavioral factor, comparable longitudinal data were unavailable across all five cohorts and complete transition samples, precluding its inclusion in the primary models.

### Statistical analysis

The primary design used complete six-node adjacent-wave pairs. For inverse probability weighting, the selection outcome was defined as complete symptom data at both wave t and wave t + 1 among candidate adjacent-wave pairs. Stabilized inverse probability weights were calculated as the numerator probability of complete transition observation divided by the denominator probability conditional on age, sex, education, chronic disease count, wave, and scaled survey weight. The scaled survey weight entered the selection model as a predictor of complete-transition observation, whereas the stabilized inverse probability weight addressed differential inclusion in the complete-transition sample. Stabilized inverse probability weights were truncated at the 1st and 99th percentiles within each cohort and multiplied by scaled survey weights for the CLPN models. The resulting analytical weight thus combined distinct sampling-representation and selection-adjustment components; the target estimand was an average conditional association in the survey-weighted complete-transition population, not a fully design-based national estimate. Missing covariate values used in the weighting and outcome models were imputed within each cohort using medians for continuous variables and modes for binary sex. Because the primary IPW model did not incorporate lagged symptoms, prior missingness patterns, prior interview status, or functional health measures, and because we did not implement outcome-specific transition samples or multiple imputation, residual selection cannot be excluded, particularly in SHARE, where complete-transition observation was lower.

#### Cross-lagged panel network estimation

For each cohort and each symptom node at wave t + 1, we fitted a weighted logistic regression model that included all six symptoms at wave t, sociodemographic covariates, and wave indicators. This approach adhered to the CLPN principle of estimating item-level autoregressive and cross-lagged predictive pathways (16, 17). Because the network contained only six harmonized binary nodes and the study focused on effect estimation and cross-cohort comparability, we used fully adjusted regression-based CLPN models rather than penalized edge selection algorithms (18). This approach enabled direct estimation of cohort-specific odds ratios, robust standard errors, and random-effects pooled estimates for all directed edges. The coefficient for each lagged symptom was interpreted as a directed lagged predictive edge from symptom k at wave t to symptom j at wave t + 1. Autoregressive edges represented symptom persistence, whereas cross-symptom edges represented lagged predictive associations between distinct nodes. Participant-clustered robust standard errors were used because individuals could contribute multiple transitions. Because harmonized files did not provide comparable strata and primary sampling unit variables across all cohorts, scaled person-level weights and participant-clustered robust standard errors were used rather than full design-based variance estimation. While cluster-robust standard errors address dependence in precision estimates, they do not disentangle stable between-person differences from within-person symptom changes. Consequently, the estimated edges reflect between-person predictive associations conditional on prior symptoms, rather than within-person causal symptom dynamics.

#### Pooling and network metrics

Because formal guidance for aggregating symptom networks is still developing (19), cohort-specific log odds ratios were pooled for each directed edge using REML random-effects meta-analyses. Heterogeneity was summarized using I^2^ and τ^2^. Prediction intervals were calculated on the log-odds-ratio scale using the pooled estimate and estimated between-cohort variance, then exponentiated to the odds-ratio scale. Nominal statistical significance was defined as two-sided *p* < 0.05, and FDR significance was defined as a Benjamini–Hochberg-adjusted value of *q* < 0.05 across all cross-symptom edges. Exploratory centrality indices were calculated from the pooled log-OR matrix after removing autoregressive edges. Because centrality was based on conditional log-ORs from binary outcome models rather than resampled individual-level networks, centrality rankings were treated as descriptive only, consistent with cautions regarding sampling variability and stability in network analysis (20). Cross-cohort similarity was evaluated with edge-weight correlations, Frobenius distance, and Jaccard similarity for nominally significant edges; these exploratory metrics were not used to make country-level comparisons.

#### Cohort-specific and sensitivity analyses

For clinically relevant or highly heterogeneous edges, we summarized the weakest and strongest cohort-specific estimates and the number of cohorts with statistically significant higher-odds associations. We performed leave-one-cohort-out random-effects meta-analyses, including a specific exclusion of SHARE because it had the lowest complete-transition proportion. Hartung-Knapp adjustment was applied to REML random-effects models on the log-odds-ratio scale as a sensitivity analysis for cross-symptom edges. Extended covariate models were not implemented because they would require a substantially different common-variable construction, reduce cross-cohort comparability, and potentially change the analytic sample.

### Ethics

All source cohorts obtained informed consent from participants and received institutional ethics approval. This secondary analysis used de-identified public or registered-use data and involved no direct participant contact. No new primary data collection or participant contact was conducted.

### Role of the funding source

The study funders had no role in study design, data collection, data analysis, data interpretation, or writing of the report.

## Results

### Analytic sample

**Figure 1:**
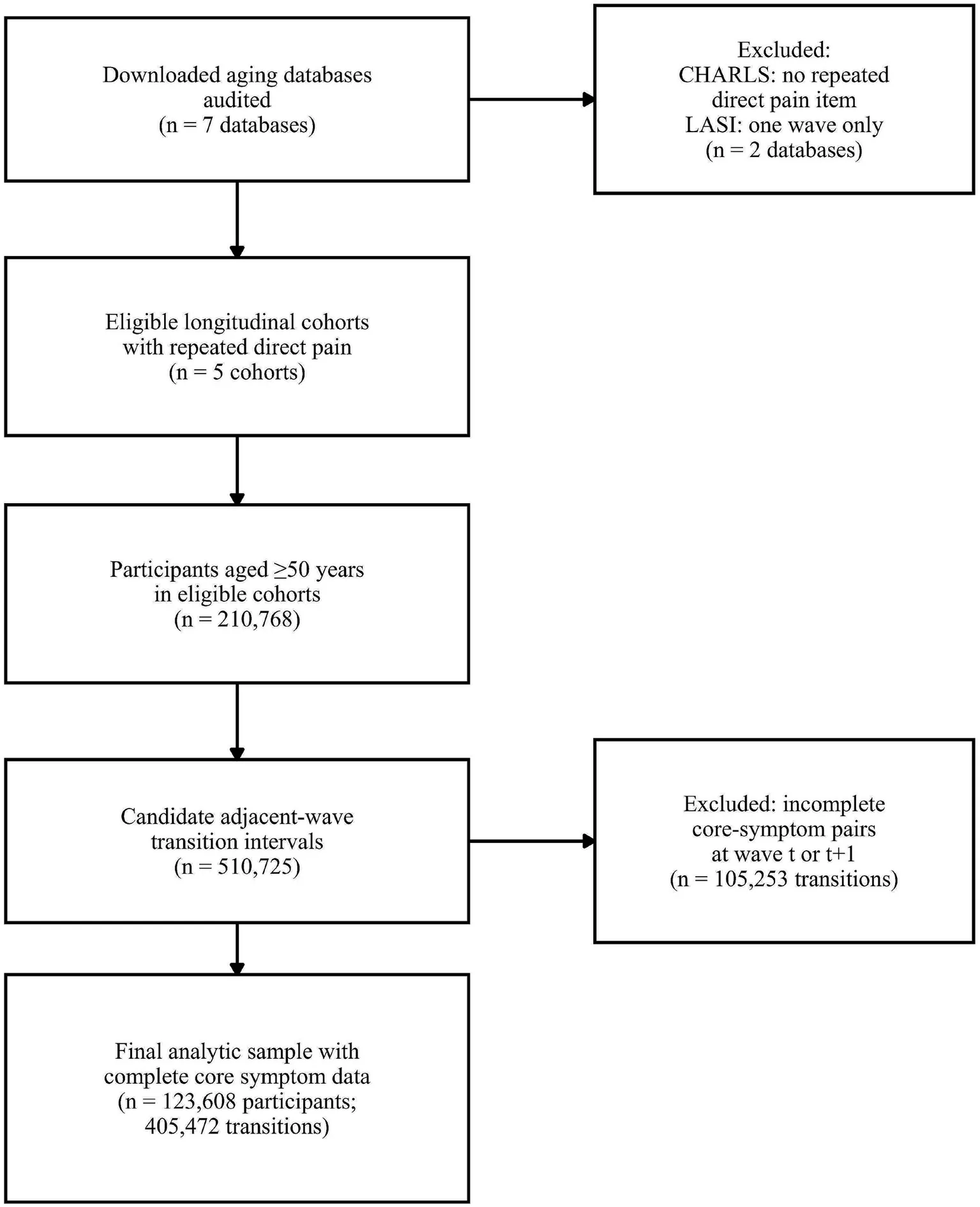
Study flow.

Across the five main cohorts, 123,608 participants contributed 405,472 complete adjacent-wave transitions out of 510,725 candidate transitions (Table 1; Figure 1). The number of analytic participants per cohort ranged from 9,611 (KLoSA) to 47,869 (HRS). Mean baseline age ranged from 59.00 to 65.68 years across cohorts, and women comprised 52.9 to 55.9% of participants. Table 1 details the cohort-specific prevalence of low education, chronic disease count, arthritis prevalence, survey waves, and transition intervals.

**Table 1:** Cohort design and baseline demographic/health characteristics.

Cohort	Country/region	Participants, *n*	Person-waves, *n*	Complete transitions, *n*	Survey waves	Transition interval	Age, mean (SE), y	Women, % (SE)	Low education, % (SE)	Chronic disease count, mean (SE)	Arthritis, % (SE)
ELSA	England	14,083	80,590	61,813	1–9	Adjacent survey waves	61.51 (0.08)	55.0% (SE 0.4)	45.9% (SE 0.4)	0.89 (0.01)	26.6% (SE 0.4)
HRS	United States	34,748	245,642	191,364	2–15	Median 2 years (IQR 2–2)	59.38 (0.06)	54.5% (SE 0.3)	10.1% (SE 0.2)	1.10 (0.01)	32.0% (SE 0.3)
KLoSA	South Korea	9,611	58,919	48,206	1–8	Median 2 years (IQR 2–2)	59.00 (0.11)	52.9% (SE 0.6)	51.7% (SE 0.6)	0.59 (0.01)	13.3% (SE 0.4)
MHAS	Mexico	17,297	60,988	39,054	1–5	Median 3 years (IQR 2–3)	59.23 (0.12)	55.9% (SE 0.8)	75.7% (SE 0.7)	0.72 (0.01)	16.3% (SE 0.6)
SHARE	Europe/Israel	47,869	157,259	65,035	5–8	Median 2 years (IQR 2–2)	65.68 (0.08)	54.7% (SE 0.4)	33.8% (SE 0.4)	1.37 (0.01)	37.6% (SE 0.4)

Low education was derived using cohort-specific harmonized education scales and is presented descriptively because education coding is not directly comparable across cohorts. Models estimated wave-to-wave temporal predictive associations rather than fixed-duration effects; survey intervals varied across cohorts.

### Symptom prevalence and co-occurrence

At the baseline wave (*t*) among complete transitions, weighted prevalence ranged from 30.8 to 55.0% for pain, 29.5 to 40.3% for sleep disturbance, and 14.6 to 43.0% for depressed mood. Corresponding ranges were 12.2 to 31.6% for loneliness, 21.4 to 36.3% for fatigue/effort, and 11.2 to 35.5% for low positive affect (Table 2). In KLoSA, low positive affect remained more prevalent than in other cohorts even after applying the conservative very-rarely-happy threshold. Results involving low positive affect were therefore interpreted cautiously, and an additional five-node sensitivity network excluding this node was analyzed.

**Table 2:** Lag-wave symptom-node prevalence among complete adjacent-wave transitions by cohort.

Cohort	Pain, % (SE)	Sleep disturbance, % (SE)	Depressed mood, % (SE)	Loneliness, % (SE)	Fatigue/effort, % (SE)	Low positive affect, % (SE)
ELSA	37.7% (SE 0.4)	40.3% (SE 0.4)	16.1% (SE 0.3)	12.2% (SE 0.3)	21.4% (SE 0.3)	11.2% (SE 0.3)
HRS	30.8% (SE 0.3)	32.2% (SE 0.3)	14.6% (SE 0.2)	15.2% (SE 0.2)	24.3% (SE 0.3)	13.0% (SE 0.2)
KLoSA	55.0% (SE 0.6)	29.5% (SE 0.5)	25.8% (SE 0.5)	27.0% (SE 0.5)	32.2% (SE 0.5)	35.5% (SE 0.6)
MHAS	40.1% (SE 0.8)	37.2% (SE 0.8)	34.9% (SE 0.7)	31.6% (SE 0.7)	34.6% (SE 0.7)	23.2% (SE 0.7)
SHARE	46.9% (SE 0.4)	35.6% (SE 0.4)	43.0% (SE 0.4)	13.0% (SE 0.3)	36.3% (SE 0.4)	13.7% (SE 0.3)

Values represent weighted prevalence of symptoms at wave t among complete adjacent-wave transitions.

### Pooled lagged symptom network

**Figure 2:**
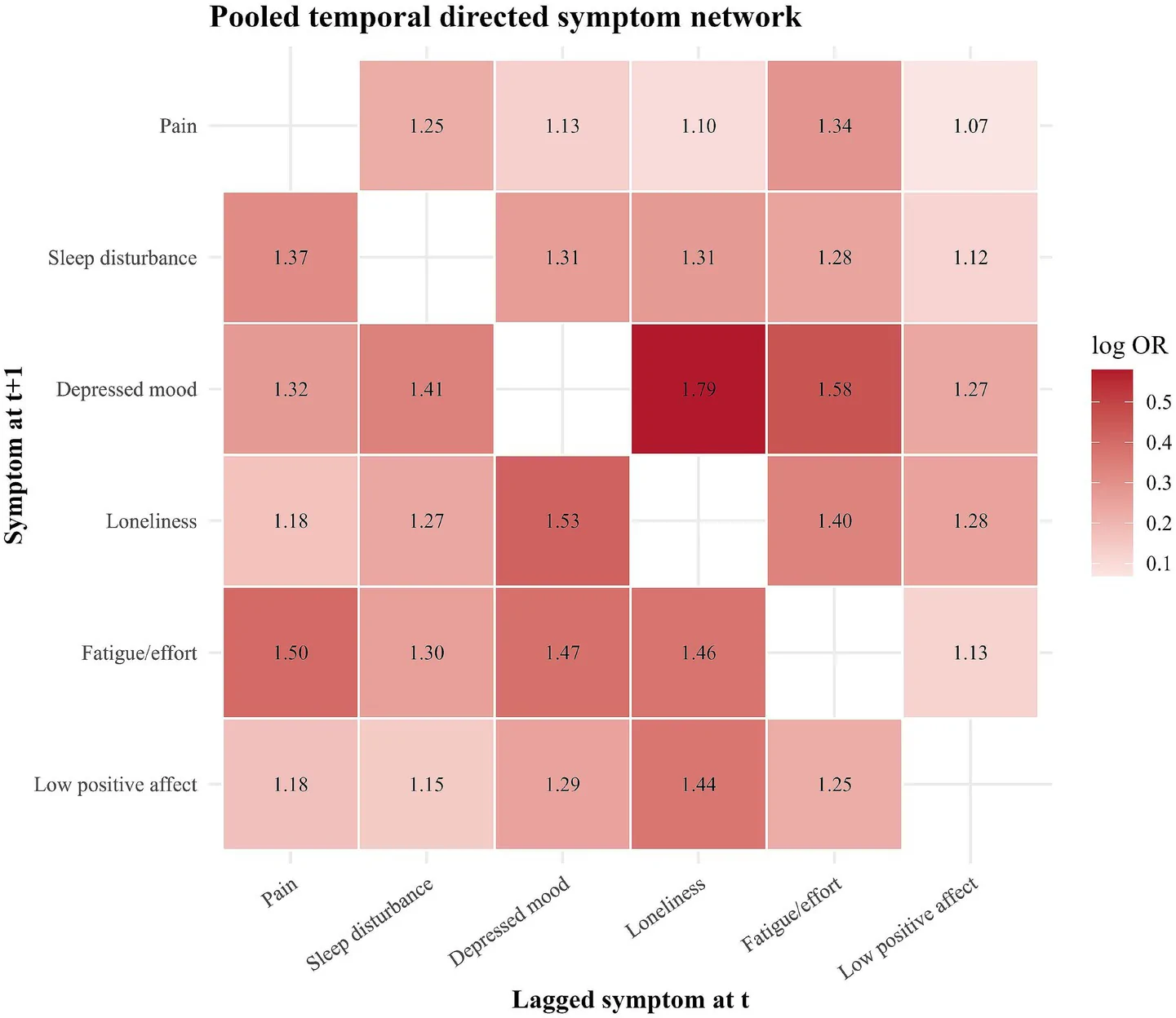
Pooled lagged associations.

**Figure 3:**
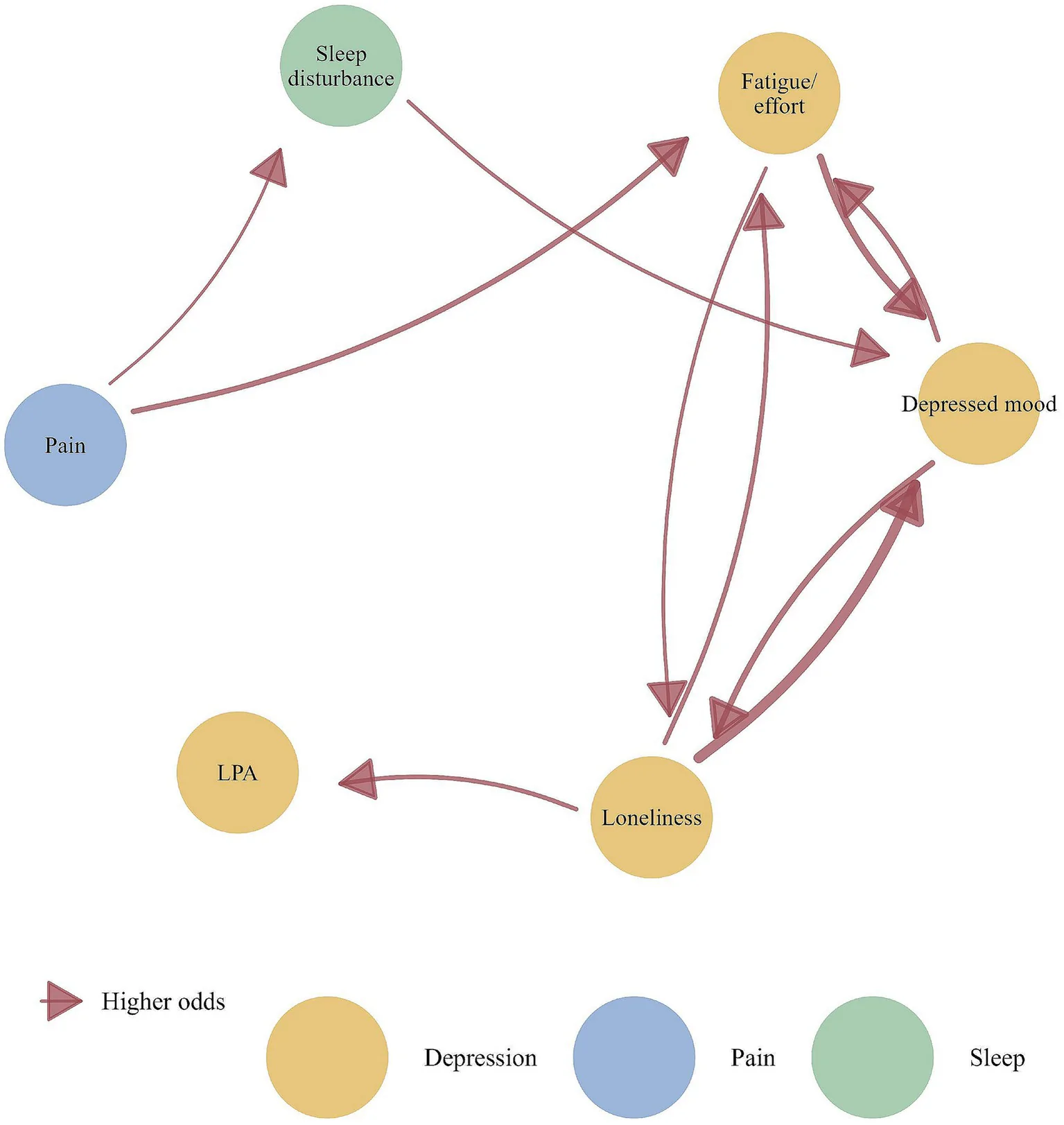
Directed symptom network.

In the pooled cross-symptom network, all leading temporal edges had ORs above 1, reflecting positive lagged predictive associations with higher odds after adjustment for autoregression, other lagged symptoms, covariates, wave indicators, survey weights, and inverse probability weights (Figures 2, 3; Table 3). Table 3 presents the cross-symptom edges with the lowest FDR *q* values. Table 4 focuses on key clinically relevant pathways, reporting prediction intervals and Hartung–Knapp sensitivity inference. The most statistically precise pooled cross-symptom association was sleep disturbance predicting later depressed mood (OR 1.41 (1.36–1.46); FDR q = 5.17e-72; I^2^ = 43.3%). Although this was not the largest effect estimate, loneliness predicting depressed mood yielded a larger pooled OR (1.79 (1.58–2.01)) but greater heterogeneity. Fatigue/effort predicted later sleep disturbance (OR 1.28 (1.21–1.35); FDR *q* = 3.92e-20), while sleep disturbance predicted later fatigue/effort (OR 1.30 (1.23–1.37); FDR *q* = 1.37e-20).

**Table 3:** Strongest pooled cross-symptom temporal edges.

Directed edge	Pooled OR (95% CI)	FDR q	I^2^, %	τ^2^
Sleep disturbance to depressed mood	1.41 (1.36–1.46)	5.17e-72	43.3	7.43e-04
Sleep disturbance to fatigue/effort	1.30 (1.23–1.37)	1.37e-20	76.2	0.003
Loneliness to depressed mood	1.79 (1.58–2.01)	2.13e-20	92.5	0.017
Fatigue/effort to sleep disturbance	1.28 (1.21–1.35)	3.92e-20	70.8	0.002
Sleep disturbance to loneliness	1.27 (1.20–1.34)	4.80e-18	66.4	0.002
Loneliness to fatigue/effort	1.46 (1.33–1.60)	2.00e-15	87.4	0.009
Fatigue/effort to loneliness	1.40 (1.29–1.52)	2.00e-15	83.5	0.007
Depressed mood to fatigue/effort	1.47 (1.34–1.62)	9.49e-15	89.2	0.01
Fatigue/effort to depressed mood	1.58 (1.38–1.81)	1.63e-10	95.4	0.023
Pain to depressed mood	1.32 (1.20–1.44)	2.88e-09	90.2	0.009

**Table 4:** Key clinically relevant lagged symptom-level associations with REML prediction intervals and Hartung-Knapp sensitivity inference.

Directed edge	REML pooled OR (95% CI)	95% prediction interval	I^2^, %	Hartung-Knapp OR (95% CI)	Hartung-Knapp *P*	Significant cohorts, n/5
Sleep disturbance to depressed mood	1.41 (1.36–1.46)	1.32–1.50	43.3	1.41 (1.33–1.49)	8.28e-05	5/5
Loneliness to depressed mood	1.79 (1.58–2.01)	1.35–2.37	92.5	1.79 (1.50–2.12)	0.000732	5/5
Fatigue/effort to depressed mood	1.58 (1.38–1.81)	1.14–2.19	95.4	1.58 (1.30–1.92)	0.00295	5/5
Pain to depressed mood	1.32 (1.20–1.44)	1.07–1.61	90.2	1.32 (1.16–1.49)	0.00343	5/5
Pain to sleep disturbance	1.37 (1.21–1.55)	1.01–1.85	96.8	1.37 (1.15–1.63)	0.00786	5/5
Depressed mood to sleep disturbance	1.31 (1.13–1.51)	0.93–1.84	95.5	1.31 (1.07–1.60)	0.0208	5/5
Sleep disturbance to pain	1.25 (1.12–1.39)	0.98–1.60	94.2	1.25 (1.08–1.45)	0.0136	5/5
Pain to fatigue/effort	1.50 (1.30–1.72)	1.06–2.10	96.9	1.50 (1.23–1.83)	0.00498	5/5
Fatigue/effort to pain	1.34 (1.21–1.48)	1.05–1.70	92.3	1.34 (1.16–1.54)	0.00499	5/5

Hartung-Knapp *P* values test the average pooled association, whereas prediction intervals describe the expected range of effects in a future comparable cohort. Edges with prediction intervals crossing 1.00 should not be interpreted as uniformly generalizable across cohorts.

### Pain-related pathways

Pain exhibited several cross-domain lagged associations. Specifically, baseline pain predicted later depressed mood (OR 1.32 (1.20–1.44); FDR q = 2.88e-09) and fatigue/effort (OR 1.50 (1.30–1.72); FDR q = 6.73e-08). Pain also predicted later sleep disturbance (OR 1.37 (1.21–1.55); FDR q = 1.99e-06) and loneliness (OR 1.18 (1.09–1.27); FDR q = 2.64e-05). In the reverse direction, fatigue/effort predicted later pain (OR 1.34 (1.21–1.48); FDR q = 7.41e-08), and sleep disturbance predicted later pain (OR 1.25 (1.12–1.39); FDR q = 5.43e-05).

### Cross-cohort heterogeneity

**Figure 4:**
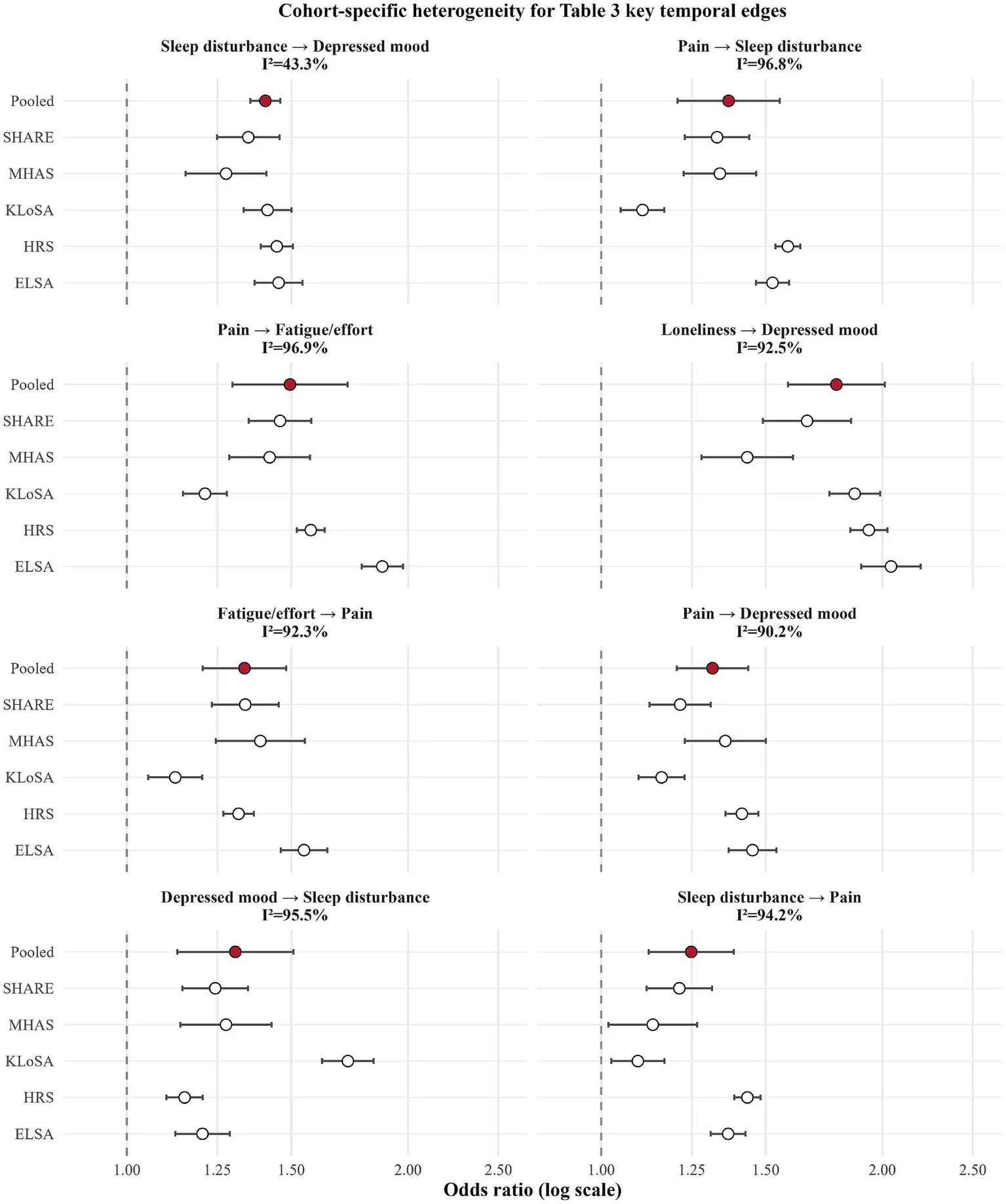
Key-edge heterogeneity.

Most leading pooled edges showed moderate-to-high heterogeneity, most notably pain predicting sleep disturbance (I^2^ = 96.8%), pain predicting fatigue/effort (I^2^ = 96.9%), loneliness predicting depressed mood (I^2^ = 92.5%), and fatigue/effort predicting depressed mood (I^2^ = 95.4%). Pooled estimates should therefore be interpreted as average cross-cohort associations rather than single common effects. Edge-weight correlations indicated the greatest similarity between ELSA and HRS (*r* = 0.88) and the lowest similarity between HRS and KLoSA (*r* = 0.22). Table 4 and Figure 4 report clinically relevant temporal edges pooled ORs, prediction intervals, I^2^ statistics, Hartung–Knapp adjusted ORs and *p* values, counts of cohorts with statistically significant associations, and cohort-specific estimates.

### Cohort-specific interpretation and sensitivity analyses

Post-estimation quality-control checks for high-heterogeneity edges did not identify obvious sparse-cell problems or model-refitting inconsistencies (Supplementary Table S5). Predictive associations for pain predicting sleep disturbance, pain predicting fatigue/effort, and loneliness predicting depressed mood were consistently positive in all five cohorts, although effect sizes varied substantially. Cohort-specific ranges were OR 1.11 (95% CI 1.05–1.17) in KLoSA to OR 1.58 (95% CI 1.54–1.63) in HRS for pain predicting sleep disturbance, and OR 1.21 (95% CI 1.15–1.28) in KLoSA to OR 1.88 (95% CI 1.78–1.98) in ELSA for pain predicting fatigue/effort. For loneliness predicting depressed mood, the range was OR 1.43 (95% CI 1.28–1.60) in MHAS to OR 2.04 (95% CI 1.90–2.20) in ELSA. Because SHARE had the lowest complete-transition proportion, we repeated the leave-one-out pooling after excluding SHARE. Key edges remained statistically significant after SHARE exclusion. Estimates were OR 1.42 (95% CI 1.38–1.47) for sleep disturbance to depressed mood, OR 1.38 (95% CI 1.17–1.62) for pain predicting sleep disturbance, OR 1.50 (95% CI 1.25–1.80) for pain predicting fatigue/effort, OR 1.81 (95% CI 1.57–2.10) for loneliness predicting depressed mood, and OR 1.34 (95% CI 1.17–1.52) for fatigue/effort predicting pain. Alternative weighting specifications preserved both directionality and statistical significance for all key edges.

### Additional sensitivity analyses

A five-node CLPN sensitivity analysis excluding the low positive affect node preserved the direction and FDR significance of the key pathways. Estimates were OR 1.41 (95% CI 1.35–1.48) for sleep disturbance predicting depressed mood and OR 1.37 (95% CI 1.21–1.56) for pain predicting sleep disturbance. Estimates were OR 1.50 (95% CI 1.30–1.73) for pain predicting fatigue/effort, OR 1.85 (95% CI 1.61–2.12) for loneliness predicting depressed mood, and OR 1.34 (95% CI 1.21–1.49) for fatigue/effort predicting pain. Lagged total symptom burden sensitivity models yielded similar estimates for pain predicting sleep disturbance, OR 1.38 (95% CI 1.22–1.55), pain predicting fatigue/effort, OR 1.50 (95% CI 1.30–1.72), and loneliness predicting depressed mood, OR 1.81 (95% CI 1.58–2.08). Covariate missingness was low across complete symptom transitions, peaking at 1.8% for education in ELSA. Complete-covariate-case analyses produced similar estimates for pain predicting sleep disturbance (OR 1.37, 95% CI 1.21–1.56), pain predicting fatigue/effort (OR 1.50, 95% CI 1.30–1.72), and loneliness predicting depressed mood (OR 1.78, 95% CI 1.58–2.01). These sensitivity analyses addressed selected robustness concerns, but they do not replace within-between, outcome-specific-sample, or time-interval stability analyses.

### Exploratory network summaries

**Figure 5:**
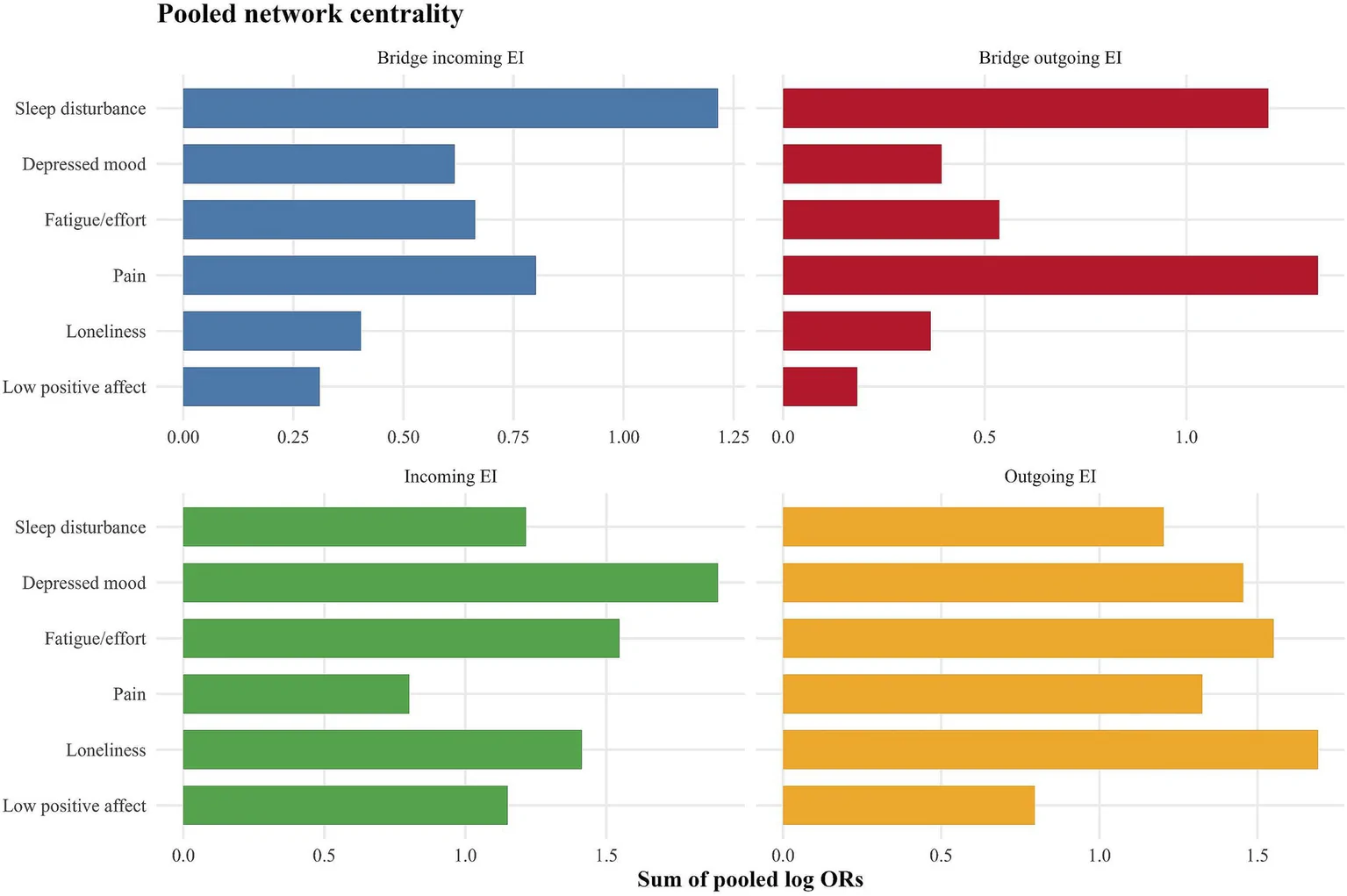
Exploratory node centrality.

Descriptive centrality metrics were calculated from the pooled log-OR matrix after removing autoregressive edges and are reported strictly as exploratory summaries (Figure 5). Because log-ORs from separate binary outcome models are not fully interchangeable and symptom domains were unequal in size, bridge centrality was not used as a primary clinical outcome. Loneliness and fatigue/effort exhibited relatively strong outgoing expected influence, whereas depressed mood demonstrated relatively strong incoming expected influence. These rankings must be interpreted cautiously and do not indicate bootstrap-stable individual-level symptom importance.

### Inverse probability weighting diagnostics

Complete symptom-transition proportions were 94.1% in ELSA, 90.3% in HRS, 99.3% in KLoSA, 86.4% in MHAS, and 46.7% in SHARE. Median truncated stabilized IPWs ranged from 0.51 to 1.00, and 1st to 99th percentile ranges are reported in Supplementary Table S2. SHARE exhibited the lowest complete-transition proportion and the widest upper tail of the truncated IPW distribution, highlighting greater potential vulnerability to selection bias despite weighting adjustment. Inverse probability weighting addresses only measured predictors of complete-transition observation; consequently, residual selection bias related to unmeasured health deterioration or symptom severity cannot be ruled out. Because person-level weights were applied to repeated transition intervals, the estimand is best interpreted as an average association across observed complete transitions rather than a strictly participant-representative national estimate.

## Discussion

In this international longitudinal multi-cohort study of adults aged ≥ 50 years, pain, sleep disturbance, loneliness, fatigue/effort, depressed mood, and low positive affect were interconnected through prospective lagged predictive associations, although the effect of magnitudes varied substantially across cohorts. Because SHARE was analyzed as a single cohort, these findings should reflect cross-cohort variability rather than direct country-level evidence. Sleep disturbance showed the most statistically consistent pooled lagged predictive association with later depressed mood. Furthermore, baseline pain showed later depressed mood, fatigue/effort, sleep disturbance, and loneliness, and was itself associated with later fatigue/effort and sleep disturbance. While these findings support a reproducible pattern of lagged symptom co-occurrence, they do not establish within-person causal symptom dynamics.

The sleep-to-depressed-mood association was the most statistically precise cross-symptom edge in the pooled analysis. This aligns with recent longitudinal and network-based evidence indicating that sleep and somatic symptoms precede or predict later affective symptoms (21, 22). Mechanistically, sleep disturbance may reflect several intertwined processes, including impaired emotion regulation, inflammatory and stress-system activation, heightened pain sensitivity, and reduced daytime functioning (23, 24). In addition, the bidirectional sleep-to-fatigue and fatigue-to-sleep pathways also indicate a recurrent somatic-affective pattern that may influence rehabilitation engagement and physical activity tolerance, although these intervention effects were not empirically tested in this study.

Pain predicted several subsequent symptoms across domains; however, these coefficients represent conditional rather than predictive associations and symptom-level causal effects. This finding extends prior longitudinal evidence on bidirectional pain–depression associations by mapping specific symptom-level links among sleep, fatigue, loneliness, and depressed mood. Although sleep-focused and cognitive-behavioral approaches have been evaluated for individuals living with pain (25–27), our pain-to-sleep and pain-to-fatigue findings highlight the clinical value of assessing these co-occurring domains early before depressed mood manifests at subsequent survey waves.

Loneliness showed a strong lagged predictive association with later depressed mood, consistent with geriatric evidence linking social context and subjective isolation to adverse health trajectories in later adulthood (28, 29). Furthermore, systematic reviews and meta-reviews indicate that loneliness and social isolation are modifiable risk factors in adults aged ≥ 50 years (30–32). These findings suggest that clinical assessment of pain or sleep symptoms should also consider loneliness and fatigue, while recognizing that the present study did not test intervention strategies.

Cross-cohort heterogeneity was substantial. ELSA and HRS exhibited the highest edge-weight correlation, whereas similarity was lower between HRS and KLoSA. Cohort-specific and QC analyses suggested that high I^2^ values reflected variation in effect magnitude rather than coding, model-fitting, or pooling errors. However, this heterogeneity could also reflect differences in item wording, response options, thresholding, survey intervals, sample composition, and unmeasured cohort context. Because the analysis did not disaggregate SHARE by country or model country-level moderators, heterogeneity cannot be definitively attributed to cultural, healthcare-system, or country-level factors. Nevertheless, predictive associations for pain with sleep disturbance, pain with fatigue/effort, and loneliness with depressed mood were consistently positive across all five cohorts despite considerable magnitude variation in effect. Prediction intervals and Hartung–Knapp analyses supported several key pooled pathways, although prediction intervals for certain high-heterogeneity edges crossed the null. Leave-one-cohort-out, SHARE-exclusion, five-node, lagged-burden, complete-covariate-case, and alternative-weighting analyses demonstrated that the main conclusions were not driven by a single cohort, the low positive affect node, total lagged symptom burden, covariate imputation, or the IPW specification. These pathways should therefore be interpreted as statistically supported average lagged associations with important cohort-specific effect-size variation, not as uniform country-level effects.

This study has several strengths. First, it leveraged large harmonized longitudinal cohorts while retaining granular symptom-level information. Second, it isolated sleep items from depressive symptom nodes, applied participant-clustered robust standard errors for repeated transitions, used inverse probability weighting to account for complete-transition selection, and quantified cross-cohort heterogeneity. Third, the analysis combined edge-level meta-analyses with descriptive network-level similarity metrics. Finally, it maintained full transparency regarding variable harmonization decisions, including the exclusion of CHARLS due to missing repeated direct pain measures and LASI due to single-wave availability.

Several limitations should be considered. First, symptom nodes were operationalized using self-reported single items or brief scale items rather than clinical diagnoses. Consequently, pain does not denote chronic pain, sleep disturbance does not represent clinical insomnia, depressed mood does not reflect major depressive disorder, and fatigue/effort does not indicate a clinical fatigue syndrome. Second, harmonization required mapping CES-D, EURO-D, and cohort-specific pain items onto common binary nodes. While this improved comparability, it may have reduced within-cohort measurement detail, and residual measurement non-equivalence likely contributed to between-cohort heterogeneity. The low-positive-affect node requires particular caution because KLoSA used a four-level frequency item, whereas other cohorts used binary happy or enjoyment items. Third, standard CLPN models can conflate within-person symptom changes with stable between-person differences. The present analysis did not implement a Mundlak within-between decomposition, random-intercept logistic model, or other framework separating stable person-level differences from within-person deviations. Fourth, the primary complete six-node transition design excluded transitions with missing data on specific outcomes. Outcome-specific samples, multiple imputation, and IPCW models incorporating lagged symptoms and prior missingness were not implemented in the current version. Fifth, actual elapsed survey interval information was incomplete for some harmonized sources, and predictor-by-interval or predictor-by-wave stability tests were not performed. ORs should therefore be interpreted as average adjacent-wave associations across non-identical intervals. Sixth, SHARE was analyzed as a single cohort rather than being disaggregated by country, precluding estimation of country-level differences. Seventh, random-effects meta-analyses were based on five cohorts, so heterogeneity estimates and prediction intervals may be imprecise despite Hartung–Knapp sensitivity analyses. Eighth, although survey weights and inverse probability weights were applied, complete complex survey variance estimation was limited by the availability and comparability of design variables across harmonized files. Repeated transitions weighted by person-level weights do not yield strictly national participant-level estimates. Ninth, extended covariates, such as marital status, wealth, BMI, smoking, physical activity, medication use, pain intensity, pain location, cognitive status, and other health behaviors, were not uniformly available with comparable definitions across all five cohorts and all symptom-complete transition pairs. Future studies with richer common covariate sets should evaluate whether these factors modify temporal symptom pathways (33–35).

Future studies applying random-intercept CLPN models, Mundlak within-between decompositions, or intensive longitudinal designs may clarify whether the observed associations reflect true within-person symptom dynamics. Because only five cohorts were available and no subset used identical wording and thresholds across all symptom nodes, we did not conduct a restricted random-effects meta-analysis limited to the most comparable cohorts; instead, we transparently report item-level harmonization, cohort-specific estimates, significant-cohort counts, prediction intervals, and effect ranges. In addition, variation in follow-up intervals across cohorts may also limit direct comparison of effect magnitudes.

Clinically, these results support the integrated assessment of pain, sleep disturbance, fatigue/effort, loneliness, and depressed mood in adults aged ≥ 50 years rather than a single-symptom approach. Related studies on physical activity and behavioral care provide essential clinical context for symptom assessment in adults with depressive or somatic symptoms (36–38). Although digital and behavioral studies provide examples of symptom-management approaches, the present observational analysis did not evaluate treatment effects (39). In clinical practice, adults aged ≥ 50 years presenting with pain may warrant systematic assessment for sleep disturbance and fatigue (40–42). Depressive symptoms should also be considered in this assessment context (43). Conversely, adults aged ≥ 50 years presenting with sleep disturbance may warrant assessment for co-occurring pain and fatigue before depressed mood manifests at later clinical encounters or survey waves (44, 45). These considerations are highly relevant to primary care, geriatrics, psychiatry, rehabilitation medicine, and community-based aging services, though the present observational analysis does not identify direct causal pathways or intervention targets. Future sensor-based studies should distinguish cumulative activity volume, peak intensity, temporal patterning, and fragmentation, and evaluate whether associations differ by pain site (46, 47).

In conclusion, pain, sleep disturbance, loneliness, fatigue/effort, and depressive-affective symptoms showed consistent prospective lagged associations across five international aging cohorts, though effect magnitudes varied substantially across populations. Sleep disturbance demonstrated a statistically robust lagged predictive association with later depressed mood. Collectively, these findings may inform joint symptom assessment and identify promising symptom pairs for future within-person and causal-inference research; however, they do not establish causal symptom dynamics, actionable intervention targets, or uniform country-level differences.

## Data Availability

This study analyzed harmonized and source cohort data from the Health and Retirement Study (HRS), the English Longitudinal Study of Ageing (ELSA), the Survey of Health, Ageing and Retirement in Europe (SHARE), the Korean Longitudinal Study of Aging (KLoSA), and the Mexican Health and Aging Study (MHAS), including Gateway to Global Aging Data and RAND/Harmonized data products where applicable. Access is available to registered researchers through the respective cohort or Gateway data platforms, subject to each platform’s registration procedures, data-use agreements, and access restrictions. The authors are not permitted to redistribute the underlying datasets. Derived analytic code is available from the corresponding author upon reasonable request.
