# Supplementary material for: Lagged symptom-level associations among pain, sleep disturbance, and depressive-affective symptoms in adults aged ≥50 years: an international longitudinal multi-cohort study
Source: Front Med (Lausanne). 2026 Aug 21;13:1921405. doi: 10.3389/fmed.2026.1921405 (PMC13542647; doi:10.3389/fmed.2026.1921405)
Supplement: Supplementary file 1 [file Data_Sheet_1.docx]

**Supplementary Material**

**Lagged Symptom-Level Associations Among Pain, Sleep Disturbance, and Depressive-Affective Symptoms in Adults Aged 50 Years or Older**

**Supplementary Table S1A. Symptom-node harmonization across cohorts.**

| **Node** | **ELSA** | **HRS** | **KLoSA** | **MHAS** | **SHARE** | **Coding** |
| --- | --- | --- | --- | --- | --- | --- |
| Pain | r*w painfr; direct pain-frequency/problem item | RAND r*w painfr; direct pain-frequency/problem item | r*w painfr; direct pain-frequency/problem item | r*w painfr; direct pain-frequency/problem item | r*w painfr; direct pain/problem item | Binary 0/1; direct pain item required for cohort inclusion |
| Sleep disturbance | r*w sleepr; restless sleep | RAND r*w sleepr; restless sleep | r*w sleeprl; CES-D restless sleep frequency | r*w sleepr; restless sleep | EURO-D r*w sleep | Binary 0/1; frequency-coded negative symptom present at >=2 where applicable |
| Depressed mood | r*w depres; felt depressed | RAND r*w depres; felt depressed | r*w depresl; CES-D depressed frequency | r*w depres; felt depressed | EURO-D r*w depress | Binary 0/1; frequency-coded negative symptom present at >=2 where applicable |
| Loneliness | r*w flone; felt lonely | RAND r*w flone; felt lonely | r*w flonel; CES-D lonely frequency | r*w flone; felt lonely | r*w lnlys3; 3-item loneliness mean score | Binary 0/1; SHARE loneliness mean dichotomized at >=2 |
| Fatigue/effort | r*w effort; everything was an effort | RAND r*w effort; everything was an effort | r*w effortl; CES-D effort frequency | r*w effort; everything was an effort | EURO-D r*w fatig | Binary 0/1; frequency-coded negative symptom present at >=2 where applicable |
| Low positive affect | r*w whappy; reverse-coded happy item | RAND r*w whappy; reverse-coded happy item | r*w whappyl; CES-D happy frequency | r*w whappy; reverse-coded happy item | EURO-D r*w enjoym; enjoyment item | Binary 0/1; binary happy/enjoyment items reverse-coded. KLoSA coded as 1=very rarely happy only. |

**Supplementary Table S2. Inverse probability weighting diagnostics for complete six-node transition observation.**

| **Cohort** | **Candidate transitions, n** | **Complete transitions, n** | **Complete, %** | **Median IPW** | **IPW 1st percentile** | **IPW 99th percentile** |
| --- | --- | --- | --- | --- | --- | --- |
| ELSA | 65,708 | 61,813 | 94.1 | 1.00 | 0.96 | 1.08 |
| HRS | 211,937 | 191,364 | 90.3 | 0.98 | 0.92 | 1.38 |
| KLoSA | 48,537 | 48,206 | 99.3 | 1.00 | 1.00 | 1.02 |
| MHAS | 45,196 | 39,054 | 86.4 | 0.98 | 0.90 | 1.28 |
| SHARE | 139,347 | 65,035 | 46.7 | 0.51 | 0.50 | 3.12 |

**Supplementary Table S3. Five-node sensitivity analysis excluding low positive affect.**

| **Directed edge** | **Primary OR (95% CI)** | **Five-node OR (95% CI)** | **Five-node FDR q** | **Five-node I², %** | **Interpretation** |
| --- | --- | --- | --- | --- | --- |
| Sleep disturbance to Depressed mood | 1.41 (1.36-1.46) | 1.41 (1.35-1.48) | 1.25e-46 | 64.2 | Direction and statistical significance were preserved after excluding low positive affect. |
| Pain to Sleep disturbance | 1.37 (1.21-1.55) | 1.37 (1.21-1.56) | 1.41e-06 | 96.8 | Direction and statistical significance were preserved after excluding low positive affect. |
| Pain to Fatigue/effort | 1.50 (1.30-1.72) | 1.50 (1.30-1.73) | 3.45e-08 | 96.9 | Direction and statistical significance were preserved after excluding low positive affect. |
| Loneliness to Depressed mood | 1.79 (1.58-2.01) | 1.85 (1.61-2.12) | 1.18e-17 | 94.6 | Direction and statistical significance were preserved after excluding low positive affect. |
| Fatigue/effort to Pain | 1.34 (1.21-1.48) | 1.34 (1.21-1.49) | 3.45e-08 | 92.5 | Direction and statistical significance were preserved after excluding low positive affect. |
| Pain to Depressed mood | 1.32 (1.20-1.44) | 1.32 (1.21-1.45) | 2.38e-09 | 90.8 | Direction and statistical significance were preserved after excluding low positive affect. |
| Depressed mood to Sleep disturbance | 1.31 (1.13-1.51) | 1.34 (1.18-1.52) | 9.87e-06 | 94.6 | Direction and statistical significance were preserved after excluding low positive affect. |
| Sleep disturbance to Pain | 1.25 (1.12-1.39) | 1.25 (1.12-1.39) | 4.09e-05 | 94.5 | Direction and statistical significance were preserved after excluding low positive affect. |

**Supplementary Table S4. Extended covariate sensitivity feasibility.**

| **Requested covariate/domain** | **Feasibility status** |
| --- | --- |
| Marital status | Not uniformly available with comparable definitions across all five cohorts and all symptom-complete transition pairs in the constructed analysis dataset. |
| Wealth | Not uniformly available with comparable definitions across all five cohorts and all symptom-complete transition pairs in the constructed analysis dataset. |
| BMI | Not uniformly available with comparable definitions across all five cohorts and all symptom-complete transition pairs in the constructed analysis dataset. |
| Smoking | Not uniformly available with comparable definitions across all five cohorts and all symptom-complete transition pairs in the constructed analysis dataset. |
| Physical activity | Not uniformly available with comparable definitions across all five cohorts and all symptom-complete transition pairs in the constructed analysis dataset. |
| Medication use | Medication classes and reference periods were not comparable across all five cohorts and symptom-complete transition pairs. |
| Pain intensity | Direct repeated pain-frequency/problem indicators were harmonized, but comparable pain-intensity scales were not uniformly available across all five cohorts and transition pairs. |
| Cognitive status | Cognitive measures differed by instrument and were not uniformly available with comparable definitions across all five cohorts and symptom-complete transition pairs. |

**Supplementary Table S5. High-heterogeneity edge QC summary and cohort-specific effect ranges.**

| **Directed edge** | **Pooled OR (95% CI)** | **95% prediction interval** | **I², %** | **Hartung-Knapp P** | **Significant cohorts** | **Cohort-specific OR range** |
| --- | --- | --- | --- | --- | --- | --- |
| Loneliness to Depressed mood | 1.79 (1.58-2.01) | 1.35-2.37 | 92.5 | 7.32e-04 | 5/5 | MHAS 1.43 (1.28-1.60) to ELSA 2.04 (1.90-2.20) |
| Pain to Fatigue/effort | 1.50 (1.30-1.72) | 1.06-2.10 | 96.9 | 0.005 | 5/5 | KLoSA 1.21 (1.15-1.28) to ELSA 1.88 (1.78-1.98) |
| Pain to Sleep disturbance | 1.37 (1.21-1.55) | 1.01-1.85 | 96.8 | 0.008 | 5/5 | KLoSA 1.11 (1.05-1.17) to HRS 1.58 (1.54-1.63) |
